# South West Surgeons Club

**Published:** 1981

**Authors:** 


### Abstracts

**South West Surgical Club** were the guests of Professor Michael Brady and the Department of Surgery at Cork Regional Hospital, Wilton, Cork on 1st-2nd May 1981. The following are abstracts of papers read to the meeting.

### THE FROME EXPERIMENT – VALUE OF SCREENING FOR COLORECTAL CANCER

**D. C. Britton, Bath**

Cancer of the large bowel is the second most common malignant tumour in the Western world, with approximately 20,000 new cases registered each year in England and Wales. No improvement in the survival figures has occurred in the past 30 years, and since 1970 the number of deaths per annum due to large bowel cancer in Great Britain has been rising. The prognosis of the disease is directly related to the degree of centrifugal spread of the tumour. Patients with cancers limited to the bowel wall have a corrected 5-year survival of about 90%, whereas those with tumours with lymphatic spread have a 5-year survival of about 30%

The prognosis for early colorectal tumours is therefore good, but a method of identifying such lesions is required. If early tumours bleed the detection of occult blood in the faeces may be a valuable screening test for colorectal cancer. This paper describes the results of screening in a population of 8925 persons for cancer of the colon.

2439 patients accepted the invitation. 12 tumours of the bowel were identified. 39 patients had a false-positive result, but all of these became negative after dietary restriction. One false-negative result has occurred so far.

### TECHNIQUES IN COLORECTAL SURGERY

**W. O. Kirwan, Cork**

In this institution toxic dilatation of the colon is no longer managed by emergency excisional surgery but rather by the more conservative procedure of loop ileostomy and blow hole transverse colostomy described by Turnbull et al (1970). Eight cases have been treated with this technique without mortality. This series is small but since the mortality generally quoted in the literature for this complication (Goligher, 1980) approaches 30% it is significant that no patient has died in our hospital from this complication since the institution of the present conservative approach. The failure of this technique to gain wide acceptance has probably been due to technical errors in its implementation.

The use of the EEA auto-suture apparatus was also described and initial experience reported. It was pointed out that the abdominoperineal excision is becoming an uncommon procedure with a ratio of eight anterior resections to one abdominoperineal excision. The importance of irrigating the presacral space in anterior resection cases with Shirley Sump drains for four days post-operatively was emphasised and this procedure has almost entirely eliminated the troublesome complication of pelvic sepsis. In low anterior resections temporary transverse colostomy is being abandoned in carefully chosen cases but in cases where there is any difficulty with the anastomosis or where the anastomosis is to the squamous epithelium of the anal canal it is advised that a stoma should still be instituted and closed after 10 weeks.

### EXPERIENCE IN THE USE OF THE OBTURATING BALLOON COLOSTOMY (TORBAY COLOSTOMY)

**John Rickett, Torquay**

Improvements in the techniques of anastomosis and in antibiotic chemotherapy have resulted in safer resection of the left colon. Fistulae are rarely seen. Those patients at risk of leakage may be predicted. They are often old, frail and in some degree of cardio-respiratory failure. These patients benefit from a period of temporary colostomy protection of the anastomosis. One such method of protection is the use of the Obturating Balloon Colostomy (OBC) (Torbay Colostomy) in the transverse colon. This technique has the advantage over the standard loop colostomy in that surgery is not required to close the colostomy when it is no longer needed.

The results of this technique in 42 patients were described. Included in the study were patients in whom a transverse loop colostomy would otherwise have been necessary owing to the extensive nature of the surgery, frailty of the patient or difficulty with the anastomosis. The average age of the patients in the series was 70 years. The device was left in situ for an average of 20 days in 25 patients. The colostomy site fistula healed spontaneously after the device has been removed in all except 2 patients who needed only minor surgery to achieve closure. Six patients developed clinical evidence of anastomotic leakage. Of these 3 healed with conservative measures and in only 3 patients was a transverse colostomy required. There was neither morbidity nor mortality attributable to this technique when the device was proximal to the anastomosis. Colonic necrosis at the site of placement of the device was not experienced nor were there any other late problems.

The OBC is an alternative to the loop colostomy when temporary (2 to 4 weeks) protection of the anastomosis is indicated.

### THE TWO FINGER TEST

**C. P. Sames, Bath**

Digital examination of the anal canal and lower rectum is normally carried out by using a well lubricated forefinger. This is a time honoured preliminary to more detailed assessment by means of proctoscopy and sigmoidoscopy.

However it is the author's contention that an adequate assessment of the anal canal can only be made by first using the forefinger and if this is satisfactorily accepted, supplementing it with the passage of the adjacent mid-finger. Any normal, healthy anal canal will except these two fingers (alongside each other) and the hand can be pronated to and from to examine the passage in both planes. Inability to carry out this manoeuvre without discomfort indicates some degree of major or minor stenosis, be it spasm or organic fibrosis of the sphincter musculature. The passage of a solitary finger does not necessarily exclude these conditions. The inability to insert two fingers without causing pain indicates the need for dilatation. This may be by the repeated use of dilators or, if the condition is more severe, by internal sphincterotomy, pectenotomy, or manual dilatation as recommended by Lord. I have found this 'two-finger test' a most valuable adjunct to any clinical assessment of anal or rectal pathology and the planning of future management.

Therefore as summary three 'laws' can be enunciated which are as follows:

*First Law*

The painless passage of one finger does not necessarily exclude minor anal pathology.

*Second Law*

A normal healthy anal canal will accept two examining fingers without pain or difficulty.

*Third Law*

Inability to pass two fingers without discomfort or pain denotes stenosis, either spasmodic or organic, and indicates need for some form of treatment.

A plea is made for the wider recognition and use of this simple 'two finger test'.

### SURGICAL TREATMENT OF HYPERHIDROSIS IN CHILDREN

**Michael P. Brady, Cork**

In a review of a series of adult patients suffering from hyperhidrosis and treated by upper thoracic sympathectomy; one third of those replying to a questionnaire stated that they recalled having symptoms for as long as they could remember. As a result of this finding and with the cooperation of paediatric colleagues, a small series of five patients with ages ranging from 5 to 14 years was investigated and treated by sympathectomy.

The condition in this group of young patients was severe and as socially disabling as in their adult counterparts. At play they were avoided by other children and difficulty was encountered in doing school work owing to smudging of exercise books.

The operations were done using a standard supra clavicular approach removing the thoracic sympathetic chain from T1 to T3 ganglia inclusive. This procedure was surprisingly uncomplicated and indeed, if anything, rather easier than in the adult. All children and their parents were very satisfied with the results.

A review of the literature revealed that the use of sympathectomy for hyperhidrosis in this age group had not been previously reported.

The author can confidently recommend this approach in children and these cases seem to be present in significant numbers in the community.

### DIAGNOSIS AND MONITORING IN ARTERIAL SURGERY

**R. N. Baird, Bristol**

Recent advances in ultrasound technology have led to a new pathophysiological measurements for patients with arterial diseases. Early athero-sclerosis has been detected as stiffening of the arterial wall by real-time ultrasound scanning. Digital pulse volume recordings (PVR) have identified a subgroup of the population of habitual cigarette smokers with an exaggerated vascular response to the inhalation of smoke. In assessing the significance of incomplete iliac artery stenosis, femoral artery Laplace transform damping of Doppler signals appears to be more sensitive than air-filled thigh cuff pressure and PVR measurements. In the field of carotid artery disease, ultrasound imaging has been used to screen patients for arteriography particularly when the symptoms of transient cerebral ischaemia are atypical. Intraoperative monitoring of lower limb reconstructions with the PVR has led to the early detection of less-than-ideal distal limb perfusion so that the cause can be dealt with before the operative wound is closed. The ultrasound imaging systems have been used to identify post-operative changes in implanted grafts which are associated with late graft failure. Overall, this new technology has resulted in a more precise assessment of the extent of arterial disease as well as monitoring the results of treatment.

### OVERALL BENEFIT FROM LOWER BYPASS OPERATIONS

**Joseph A. O'Donnell and Michael P. Brady, Cork**

Published results of lower extremity by-pass operations usually include patient groups determined by severity of symptoms, arteriographic runoff, or use of a specific by-pass conduit. We have agressively pursued by-pass revascularisation of severely ischaemic lower extremities in all patients who can be rehabilitated and in whom any distal vascular segment was patent. This report includes every procedure performed during the period of study.

Between February 1979 and February 1981, sixty femoro-popliteal (FP) or femoro-distal (FD) bypass procedures were performed using 30 autogenous saphenous veins (ASV), 29 polytetrafleuroethylene (PTFE) conduits and one composite of ASV and dacron (Table 1).

Most patients (77%) were between 60/79 years old and were male (90%). All patients had ischaemic rest pain, and/or necrosis. Analysis of results was for mortality, patency by the life table method, and limb loss after bypass failure.

One patient died of a myocardial infarction for an operative mortality of 1.7%. Patency at two years was 43% for ASV and 41% for PTFE. Extremity loss occurred in 75% (9/12) of early failures (< one month). In late failures (> one month), however, extremity loss was 20% (3/15).

Patency of ASV and PTFE were comparable but not encouraging. Late failure (> one month) however, did not result in extremity loss in most cases, while early failure (< one month) usually resulted in loss of the extremity. Ulceration and/or gangrene was frequently present and digital amputation frequently necessary (20%). In these cases the extremity was saved if patency was maintained during healing. Thus many bypasses which failed had been invaluable in limb preservation. Three patients had bifurcation grafts to two tibial vessels while an arteriovenous fistula was constructed in five extremities to improve patency. We continue to recommend bypass reconstruction in severely ischaemic extremities even with suspect arterial runoff in anticipation of moderately good patency, but very good limb salvage even in the face of late bypass failure.

### PRESSURE PROFILES AT THE PYLORUS

**P. Gaffney, Cork**

In 1973 Fisher et al demonstrated a physiological sphincter at the pylorus which they claimed was abnormal in patients with gastric ulceration. Subsequent investigators notably Kaye et al 1976 were unable to confirm this finding of a tonic sphincter, prompting the editors of Gastroenterology to publish an article 'The Fickle Pylorus', in which the author attempts to explain the divergent results on differences in methodology, namely Fishers group were studied in the right lateral position, Kaye's in the supine position. 20 subjects had a manometric study of their pylorus performed. Of 170 pressure studies 90 (53%) showed the presence of a high pressure zone (HPZ) at the pylorus.

More studies showed a HPZ in the supine than in the right lateral position. Evidence is presented which indicates that this HPZ is not the result of a tonic muscle sphincter, and that it may function to prevent premature gastric emptying.

### HYPERPARATHYROIDISM – PRIMARY AND TERTIARY: A PERSONAL SERIES OF 54 CASES

**H. J. O. White, Bristol**

Fifteen cases of primary hyperparathyroidism included ten with renal calculi and one each with pancreatitis, headaches, depression, bone cysts and nephrocalcinosis. Neck exploration revealed in thirteen a single adenoma, one had four gland hyperplasia and one two normal glands only despite re-operation and total thyroidectomy for occult carcinoma of the thyroid. Severe post-operative hypocalcaemia only occurred in the one patient with four gland hyperplasia.

Thirty-nine patients presented with tertiary disease, all in renal failure, thirty-four being on dialysis, three in renal failure but not yet on dialysis, and two with successfully functioning renal transplants. All had uncontrolled persistent hypercalcaemia, complicated by either bone or muscle pain, or in two bone disease with stress fractures. Neck exploration found all four glands in twenty-six, five glands in two patients, three glands in eleven and the remaining three two glands only. Thirty-seven of these patients histologically had hyperplastic glands and two only adenomata. Persistent hypercalcaemia required re-exploration of the neck in one patient in whom a fifth hyperplastic gland was removed.

Intra-venous methylene blue helped on two occasions out of three to identify a primary tumour where it could not otherwise be found, and on three occasions out of five in tertiary disease. There was no incidence of recurrent laryngeal nerve palsy at the initial exploration of the neck in either the primary or tertiary cases.

### 'CORK DISCS'

**T. F. Buckley, Cork**

Series of 339 patients operated upon in 7 year period (1971-78). Half of patients operated upon on basis of clinical criteria and without myelography. Some patients had multiple disc explorations but only one disc excised. Others had multiple disc excisions, the majority having one disc explored and excised. Follow-up 8 months to 8 years.

The survey revealed that:

1. Accurate clinical assessment not superseded by contrast radiology;
2. Multiple disc exploration did not lead to increased incidence of post-operative backache;
3. Multiple disc excision associated with increased incidence of persistent symptoms.

### POST-OPERATIVE CARE OF THE BREAST

**D. A. Griffiths, S. K. Rao, J. J. Phillipps, Yeovil**

The majority of breast operations in young women are for benign breast disease, e.g. fibroadenoma, cysts, abscesses and fistulae. The cosmetic result following surgery is often as important as the patient's relief at having the breast lesion removed. The careful use of circumareolar, sub-mammary and axillary incisions improve the final appearance and frequently completely disguise the surgical incision. The normal erect position of the nipple is occasionally compromised following surgery, especially if the nipple has been explored and tight dressings applied. To reduce these problems a new nipple dressing was used following breast surgery.

Fifty women at Yeovil District Hospital undergoing surgery for benign breast disease were treated with Stomahesive breast dressings. This wound dressing was a 3" flat, circular sheet with a hole cut through the centre for the nipple. This was applied after surgery to the breast and used as the only dressing for seven to eight days. The women were encouraged to wear their brassieres immediately post-operatively to support the breast. The Stomahesive dressing adhered to the breast contours and the spare left for the nipple encouraged normal healing.

The patients were sent home and examined in the Outpatients clinic within a few days. Removal of the nipple dressing was easy and all the wounds were completely healed without evidence of haematoma or infection.

The wounds were reassessed at one month post-operation and both the operated nipple and non-operated nipple were identical in shape and size.

Fifty women were given post-operative Stomahesive breast dressings with satisfactory results for patient comfort, wound healing and cosmetic effect.

### MANAGEMENT OF URETHRAL STRICTURE

**Patrick Smith and Michael Roberts, Bristol**

Stricture of the male urethra remains a common problem but in the last ten years a changing pattern of management has been evident. The commonest cause of stricture now is following urethral instrumentation and, in particular, trans-urethral surgery. The commonest site for these strictures is in the proximal portion of the bulb of the urethra.

The traditional approach to treatment by repeated urethral dilatation has virtually disappeared in this unit. Coincident with this has been a marked reduction in the morbidity of bleeding, infection and false passage formation that was so often associated with this technique.

Urethroplasty using skin inlay techniques or, more recently, direct anastomosis of the urethra, established itself as a standard surgical treatment some ten years ago. However, the experience in Bristol over that time has suggested that these operations have a significant failure rate and carry equally significant complication rates. Furthermore, these are technically difficult procedures and often present a severe strain to the elderly male.

The third method of stricture treatment, namely urethrotomy, underwent a dramatic revival with the development of direct vision incision using Sachse optical urethrotome. This technique was first described in the United Kingdom at a previous meeting of the South West Surgeons (Bath) by one of the authors (PJBS). This has now become the treatment of choice for urethral stricture in the male. It is a simple technique for those trained in endoscopy, carries little morbidity and, to date, no mortalty. At two, three and five year follow-up reviews it has produced an average of 80% success rate in relieving stricture symptoms.

### CHRONIC RETENTION OF URINE IN WOMEN

**N. Slade and P. H. Powell, Bristol**

Chronic retention in the male patient is a well recognised complication of prostatic obstruction. Apart from the problem of renal failure which may accompany it the urologist is mainly concerned with the return of bladder tone post-operatively. Chronic retention in women is uncommon. Little is known of its aetiology but it has previously been assumed that obstruction is the cause. To this end procedures such as radical urethra dilation, bladder neck resection and V/Y urethroplasty have been adopted.

In this study we made clinical and urodynamic assessment of thirty-two females with a residual urine exceeding 400 mls (unassociated with neurological signs and symptoms). The mean length of history was 6.5 years, mean age 57 years, and 40% gave a past history of major gynaecological surgery. Only four of the thirty-two patients were found to have high pressure bladders on urodynamic investigation and only two of these four were considered to be obstructed. Procedures which lowered outflow resistance appeared to have little effect on reducing the residual urine or the incidence of urinary infection. In eighteen of the thirty-two patients treatment was considered unnecessary although six are still known to have a palpable bladder. Three patients have long term continuous bladder drainage. Eleven patients were started on a self-catheterisation regime with a very satisfactory outcome and little complication from urinary tract infection.

